# Biocompatibility of Poly-ε-caprolactone-hydroxyapatite composite on mouse bone marrow-derived osteoblasts and endothelial cells

**DOI:** 10.1186/1749-799X-4-5

**Published:** 2009-02-26

**Authors:** Haiying Yu, Paul H Wooley, Shang-You Yang

**Affiliations:** 1 Department of Biomedical Engineering, Wayne State University, Detroit, Michigan, USA; 2 Department of Orthopaedic Surgery, Wayne State University, Detroit, Michigan, USA; 3 Orthopaedic Research Institute, Via Christi Health System, Department of Biological Sciences, Wichita State University, 1845 Fairmount Street, Wichita, KS 67260, USA

## Abstract

**Background:**

Tissue-engineered bone may be developed by seeding the cells capable of both osteogenesis and vascularization on biocompatible composite scaffolds. The current study investigated the performance of mice bone marrow-derived osteogenic cells and endothelial cells as seeded on hydroxyapatite (HA) and poly-ε-caprolactone (PCL) composite scaffolds.

**Methods:**

Mononuclear cells were induced to osteoblasts and endothelial cells respectively, which were defined by the expression of osteocalcin, alkaline phosphatase (ALP), and deposits of calcium-containing crystal for osteoblasts, or by the expression of vascular endothelial growth factor receptor-2 (VEGFR-2) and von Willebrand factor (vWF), and the formation of a capillary network in Matrigel™ for endothelial cells. Both types of cell were seeded respectively on PCL-HA scaffolds at HA to PCL weight ratio of 1:1, 1:4, or 0:1 and were evaluated using scanning electron microscopy, ALP activity (of osteoblasts) and nitric oxide production (of endothelial cells) plus the assessment of cell viability.

**Results:**

The results indicated that HA led to a positive stimulation of osteoblasts viability and ALP activity, while HA showed less influence on endothelial cells viability. An elevated nitric oxide production of endothelial cells was observed in HA-containing group.

**Conclusion:**

Supplement of HA into PCL improved biocompatible for bone marrow-derived osteoblasts and endothelial cells. The PCL-HA composite integrating with two types of cells may provide a useful system for tissue-engineered bone grafts with vascularization.

## Background

One approach to tissue engineering consists of seeding appropriate cells on a biodegradable scaffold, stimulating cell growth and differentiation *in vitro*, and then implanting the engineered complex *in vivo *to achieve functional tissue [1,2]. However seeding a single cell type into a biomaterial scaffold to replace an injured tissue that consists of multiple cell types is usually inapplicable. An alternative strategy is the generation of a composite graft, which contains not only the tissue specific cell types, but also other supportive cells, such as endothelial cells (ECs) to promote vascularization of the grafts.

ECs may be incorporated into bioengineered tissue[3,4]to promote the tissue revascularization, and transportation of oxygen and nutrients. Unfortunately, differentiated ECs isolated from most tissues including aortas, dermal capillaries and umbilical veins have inadequate proliferating capacities and are less responsive to angiogenic survival factors or anti-angiogenic signals [5,6]. In contrast, bone marrow-derived endothelial progenitor cells (EPCs) possess high potential for neovascularization and reendothelialization [7]. EPCs isolated from bone marrow or peripheral blood have been observed to undergo more than 1000 division cycles [8], indicating that even the comparatively low numbers of adult EPCs may provide sufficient seeding cells for tissue engineering applications. Bone marrow stromal cells (MSCs) are multipotent stem cells originating from the bone marrow stroma, and represent a particularly promising cell source for bone tissue engineering. They can be easily harvested, expanded *in vitro *and induced to differentiate to bone-forming cells [9]. We have therefore selected MSCs as the source of osteogenic precursors for tissue engineered bone in this project. Polycaprolactone (PCL), an FDA-approved polyester commonly as drug delivery devices used in clinical practice, has been shown to be non-toxic to cells [10,11], possessing many of the desirable properties such as degradability and plasticity. Hydroxyapatite (HA) is the inorganic part of the naturally occurring bone, and is known to be both biocompatible and osteoconductive. It suggests that the addition of HA to PCL will improve the biocompatibility and osteoconductivity of the polymer [12-14]. However, the precise dose-response relationship of HA in PCL on viability and osteogenic functions of bone marrow-derived osteoblasts remains to be elucidated. Although ECs-initiating vascularization in the engineered bone is critical [15], the survival and bioactivity of EPCs-originated ECs in biomaterials of bone graft is frequently neglected. Therefore, the objective of this study was to evaluate the biocompatibility of the HA-PCL biomaterials to both bone marrow-derived osteogenic and endothelial cells.

## Methods

### Cell Culture and Induction

Bone marrow cells were obtained from male BALB/c mice (6–8 weeks of age). Low-density bone marrow mononuclear cells (MNCs) were isolated by density centrifugation over Histopaque^®^-1083 (Sigma-Aldrich, US). Cells were then cultured in flask at 37°C and 5% CO_2 _atmosphere for differentiation of osteoblasts and endothelial cells, respectively. For osteogenic cell induction, cells were cultured in complete media [16,17] consisting of DMEM supplemented with 10% fetal bovine serum (FBS) (Invitrogen, US), 10 mM β-glycerol phosphate (Sigma-Aldrich, US), 10^-4 ^M L-ascorbic acid (Sigma-Aldrich, US), and 10 nM dexamethasone (Sigma-Aldrich, US), 2 mM glutamine (Invitrogen, US), 100 U/ml penicillin (Invitrogen, US), 100 μg/ml streptomycin (Invitrogen, US). To promote the endothelial phenotype of EPCs, the mononuclear cells were plated onto flasks coated with fibronectin (Sigma-Aldrich, US) and cultured in endothelial cell basal medium-2 (Cambrex, US) supplemented with EGM-2 MV SingleQuot^® ^kit containing 5% FBS, human epidermal growth factor (hEGF), human vascular endothelial growth factor (VEGF), human insulin-like growth factor-1 (IGF-1), hydrocortisone, penicillin (Invitrogen, US), and streptomycin (Invitrogen, US). After 4 days of culture, non-adherent cells were discarded by washing with PBS. When 60% confluence was achieved, cells were subcultured.

### Cell Characterization

Immunocytofluorescence studies were performed to detect the induced endothelial phenotypes. The induced ECs were fixed in 4% paraformaldehyde, permeated with 0.01% Triton X-100 in PBS, and incubated in 1% block serum for 1 h at 37°C. The cells were then incubated for 1 hour with monoclonal antibody against either mouse VEGFR-2 or mouse vWF (Santa Cruz, US). Bound antibodies were detected by incubation with fluorescein-5-isothiocyanate (FITC)-conjugated (Jackson ImmunoResearch, US) (for VEGFR-2) or Alexa Fluor 488-conjugated (Molecular Probes, US) (for vWF) secondary antibody. The cells were examined in fluorescence microscope. 300 μl of Matrigel™ (BD Biosciences, US) mixed with 4 × 10^4 ^EPCs-derived ECs at 4°C was dispensed into a 24-well plate and incubated at 37°C until solid. Photographs of capillary-like formation were taken at 7 days of culture in normal condition.

Similar fixation, permeabilization, and blocking processes were performed on bone marrow-derived osteoblasts, followed by the incubation with anti-osteocalcin (Santa Cruz) for 1 hour, and visualization was achieved using avidin-peroxidase complex (ABC kit, Santa Cruz Biotechnology, US). Cells were counterstained with Gill's hematoxylin solution. Calcium deposit produced by osteoblasts was demonstrated using von Kossa staining. After fixation in 4% paraformaldehyde, the cells were incubated with 1% silver nitrate solution (Sigma-Aldrich, US) under ultraviolet light for 20 minutes, and unreacted silver was removed by 5% sodium thiosulfate (Sigma-Aldrich, US). The alkaline phosphatase (ALP) activity of osteoblasts was assayed using an ALP kit (Sigma-Aldrich, US). The induced osteoblasts on slides were fixed in citrate-acetone-formaldehyde solution at room temperature for 1 minute. Following incubation in alkaline-dye mixture for 15 minutes and rinsing in distilled water, the slides were counterstained with hematoxylin solution.

### Preparation of HA-PCL Scaffolds

PCL-HA scaffolds were prepared using a particulate leaching technique as described previously [18]. The HA-PCL composite at 2 different component ratios were prepared respectively, with HA (Sigma-Aldrich, US) to PCL (Aldrich, US) at 1:1 (Group A) or 1:4 (Group B) wt/wt. PCL scaffolds without HA were used as a control (Group C). In each group, NaCl particles (particle size 212–355 μm) were used to generate a controlled level of porosity in the matrix with weight ratio to PCL at 16:1 (Group A), 8:1 (Group B) and 4:1 (Group C). PCL (Mn 80000) was dissolved in tetrahydrofuran (Sigma-Aldrich, US) at 10% wt/vol for 12 hours. HA powder (≤ 40 μm particle size) and NaCl particles were mixed to homogeneity in the PCL solution, which was sonicated for 60 seconds until viscous slurry developed. Mixtures were poured into glass dishes to a thickness of 4 mm, and dried at 37°C. After evaporation of the solvent, 1.5 × 1.5 cm squares were cut out and washed in excessive distilled water to leach out the NaCl. All materials were then sterilized in 70% ethanol and dried before biological evaluation. Samples of the PCL-HA scaffolds were gold sputter coated and their morphology was observed using SEM (Hitachi S-2400, Japan) at 15 kV. Energy Dispersive X-ray (EDX) analysis was also conducted to confirm the existence of HA particles on the composite scaffolds. The atomic percentages of calcium and phosphorus were calculated.

### Cells Culture on Scaffolds

Induced osteoblasts or endothelial cells in 50 μl suspensions (3.5 × 10^6 ^cells/ml) were respectively loaded onto each scaffold in 6-well plates. The scaffolds were left undisturbed in a 37°C incubator for 3 hours to allow cells to attach to the scaffold, after which the cells-materials complex were kept in culture using the original osteogenic or endothelial media. At day 7 the samples were harvested for and biochemical evaluation.

For morphological examination, cells-materials complex were fixed with 1.5% glutaraldehyde (Fisher Scientific, US) for 30 min at 4°C. The samples were exposed to 2% osmium tetroxide (Sigma-Aldrich, US) for 30 min. Following rinse in distilled water, they were dehydrated through a graded series of ethanol (50, 70, 90, and 100%) for 2–5 min. The dehydration was completed in hexamethyl disilazane (Fluka, Germany) for 10 minutes. After air-drying and sputter coating with gold, the cells morphology on the PCL-HA scaffolds was evaluated using SEM at 10 kV.

### Assessment of Cell Viabilities and Functions on Scaffolds

For biochemistry assay, each type of cells was seeded on 30 scaffolds per group. Cell viability was evaluated by analyzing the mitochondrial activities of the cells. The Alamar Blue assay (BioSource, US) was used to determine the mitochondrial activity after 7 days of cell culture. The cells-materials complexes were washed in Phosphate-Buffered Saline (PBS) in 6-well plate. 3 ml of new conditioned media supplemented with 200 μl of Alamar blue was added to each well. Incubation was continued at 37°C, 5% CO_2_, for 3 hours. The culture medium was then transferred to a 96-well plate and read on a spectrofluorometer (excitation wavelength 530 nm, emission wavelength 590 nm). The Alamar blue absorbance/mg of DNA values was calculated for each sample.

Cell amount were determined by a fluorometric quantification of DNA in the cells-materials complexes. After the Alamar Blue assay, the cell-scaffolds were rinsed with PBS, followed by 4 times of freezing (-80°C) and thawing (37°C) cycles for 15 minutes each. The scaffolds were then homogenized in 1.4 ml of cold 10 mM EDTA solution (Sigma-Aldrich, US). The pH of the samples was adjusted to 7.0 by adding 1 M KH_2_PO_4 _prior to the addition of 1.5 ml of the 200 ng/ml Hoechst 33258 fluorescent dye (Sigma-Aldrich, US). 100 μl of supernatant sample were read with an excitation set at 350 nm and an emission at 455 nm on a spectrofluorometer. The DNA concentration in the samples was determined against a DNA standard curve that was plotted according to a series of 100 μl of calf thymus DNA (Sigma, US) in a range of concentrations from 0.15265 to 20 μg/ml. The DNA values were used to normalize the cell viability and other cell function parameters.

Production of ALP by osteogenic cells was measured using a spectrophotometer. After the previous freeze-thaw cycle and DNA assay, 50 μl of the sample was transferred to a fresh 96-well plate and 50 μl of *p*-nitrophenyl phosphate solution (Sigma, US) was added to each sample. Following incubation for 5 min at 37°C, the production of *p*-nitrophenol in the presence of ALP was measured by monitoring light absorbance at 405 nm. The measurement of the ALP assay was normalized against the amount of total DNA in each sample.

The nitric oxide generated by endothelial cells on scaffolds was assessed using the Nitric Oxide Colorimetric Assay kit (Calbiochem, Germany) in accordance with the manufacturer's instructions. The presence of nitric oxide in the culture media of endothelial cells-materials complex was determined by detecting the colored product spectrophotometrically. The absorbance was read at 540 nm. The nitric oxide concentrations for samples were calculated according to the standard curve. Cellular nitric oxide amount was normalized by total DNA each sample.

### Statistical Analysis

All experiments were replicated three times to ensure the reproducibility, and all data was presented as the mean ± standard deviation. Single factor analysis of variance (ANOVA) with a *post hoc *LSD from SPSS™ (Student Version 10.0.5, Chicago, IL) was used to assess the statistical significance among groups, which was defined as *p *< 0.05.

## Results

### Cells Culture and Induction

**Figure 1:**
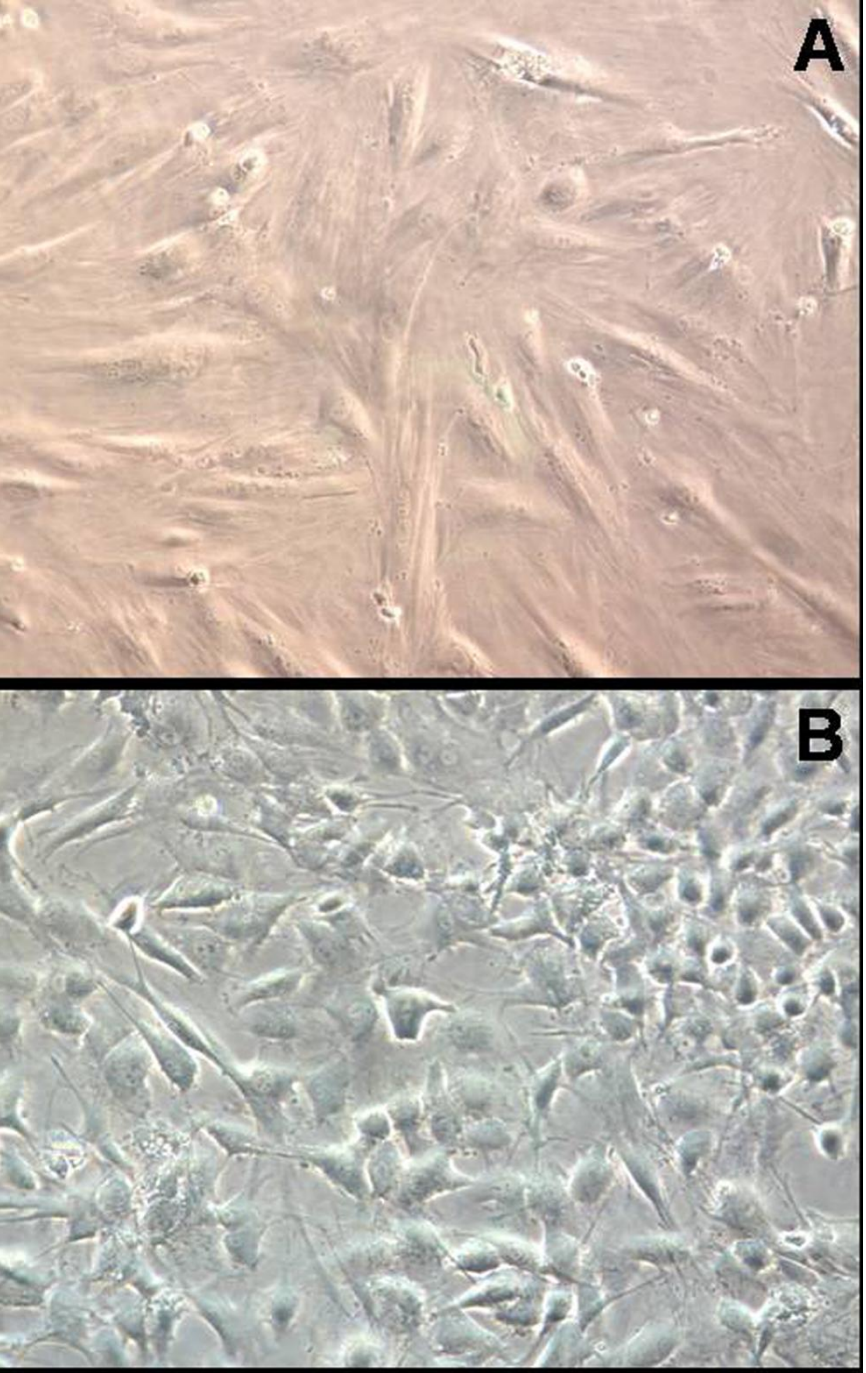
**Morphology of osteoblasts and endothelial cells**. During the 2nd passage, the mice bone marrow-derived mononuclear cells differentiated to osteoblasts exhibiting the spindle morphology (Panel A), or differentiated to endothelial cells presenting the typical cobblestone morphology (Panel B). Magnification 200×.

Cell colonies were detected in the primary passage using culture conditions with either osteogenic medium or endothelial medium. These cells differentiated and proliferated, and gradually exhibited homogeneous and specific cell morphologies. The differentiated cells displayed osteoblasts-like spindle morphology in osteogenic medium (Figure 1A), while endothelial cells presented typical cobblestone morphology (Figure 1B). These cells retained stable morphologies for more than 5 passages. We choose the 2^nd ^passage osteoblasts and endothelial cells for subsequent cell characterization and the investigation of biocompatibility.

### Cell Characterization

**Figure 2:**
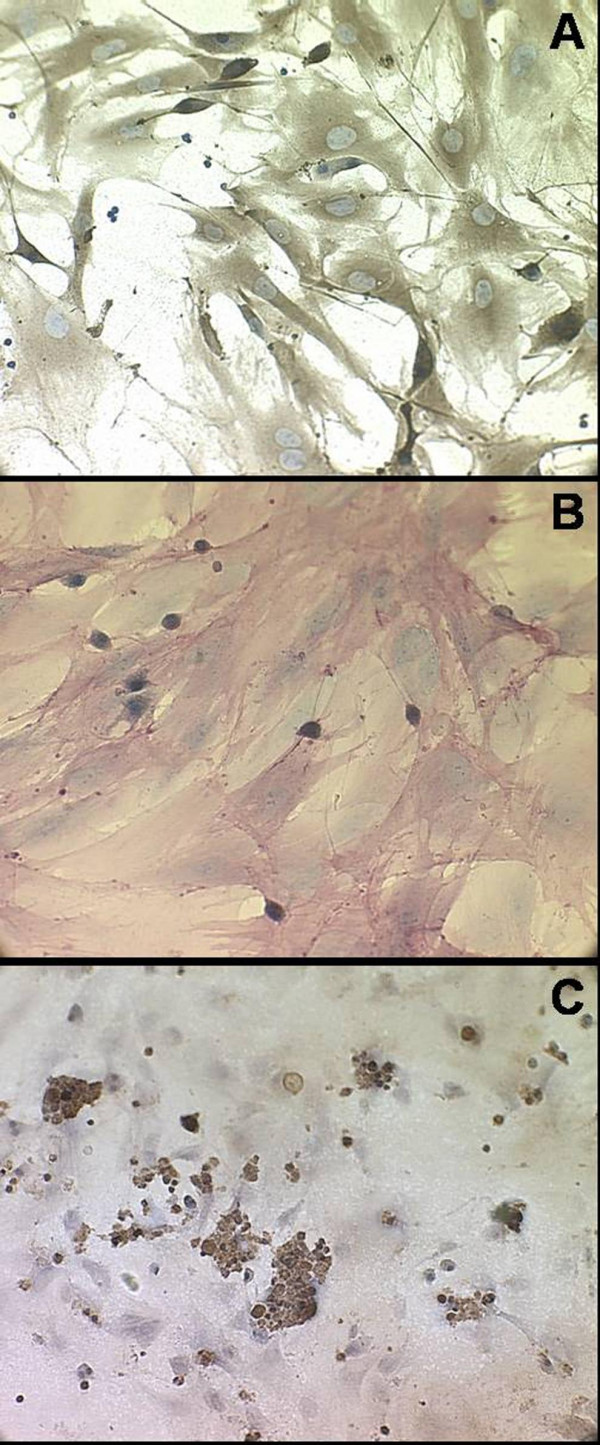
**Characterizations of osteoblasts**. Over 95% of induced osteoblasts expressed osteocalcin visualized by immunocytochemistry stains (Panel A, 200×). Alkaline phosphatase (ALP) activity of osteoblasts was assayed using an ALP kit and visualized as the pink color (Panel B, 200×). In addition, von Kossa staining was performed to reveal ossification nodules in the culture dishes of induced cells, as an indicator of osteoblasts function (Panel C, 200×).

**Figure 3:**
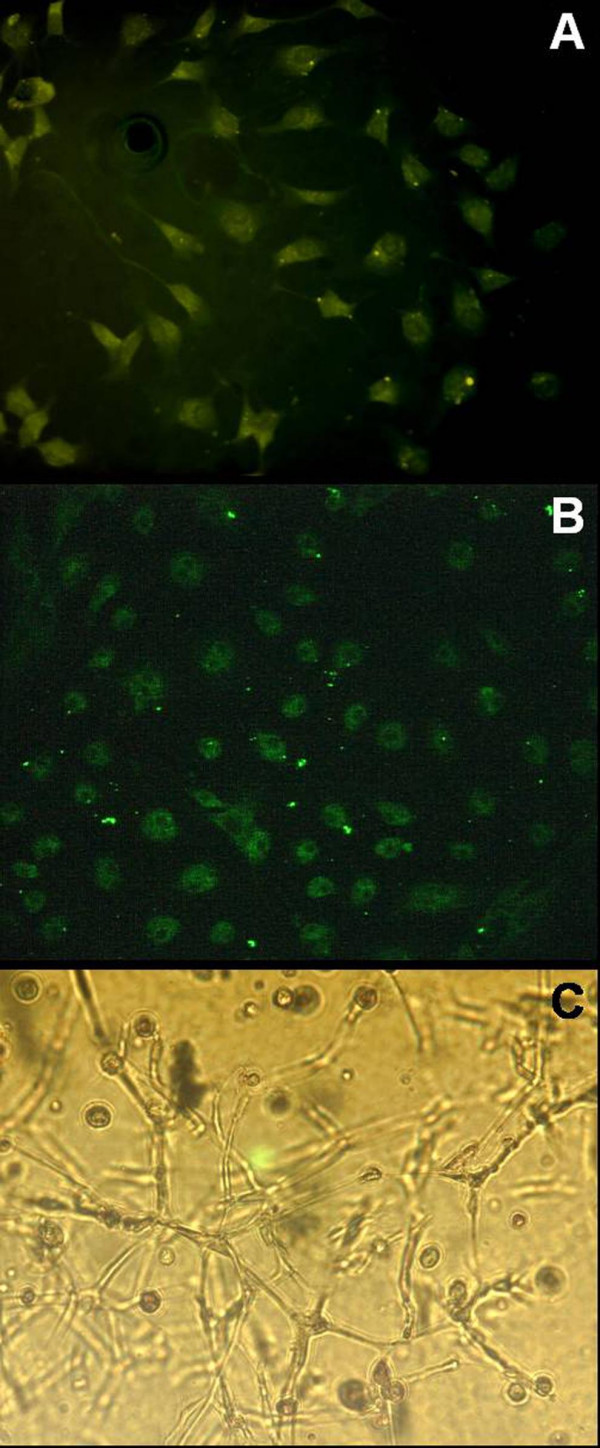
**Characterization of endothelial cells**. Panel (A) illustrated the VEGFR-2 expression on the induced ECs (200×). Over 95% of cells lighted up in dark-field microscope for cytoplasmic vWF following cultures in EC conditional medium (Panel B, 200×). After one-week incubation in a Matrigel™ basement membrane system, these cells proliferated and developed capillary-like 3-D structures (Panel C, 200×), suggesting functional endothelial phenotype.

The capacity of the induced osteoblasts to express osteocalcin was examined by immunocytochemistry (IHC) (Figure 2A). The expression of osteocalcin was detected in over 95% osteogenic-wise induced cells. These cells were also identified by positive staining for ALP (Figure 2B), which indicated that the induced cells possessed distinguishable osteoblastic phenotype. To demonstrate the ability of cells to mineralize matrix, cells cultured on Petri dishes were subjected to von Kossa stain to reveal calcium deposition (Figure 2C) where the darkly stained mineralized nodule were visualized by silver nitrate, indicating normal osteoblasts function in conditioned culture. Differentiation ability of ECs induced from bone marrow was determined by the expression of endothelial markers, VEGFR-2 and vWF, using immunocytofluorescence. The 95% endothelial-wise-induced cells expressed VEGFR-2 and vWF at 2^nd ^passage of culture (Figure 3A, B), indicating the induced cells having normal endothelial phenotype. Matrigel™ culture was performed to monitor the capability of capillary formation. Three-dimensional capillary-like networks from EPCs-derived endothelial cells were clearly established at one-week incubation (Figure 3C).

### Cells Culture on Scaffolds

**Figure 4:**
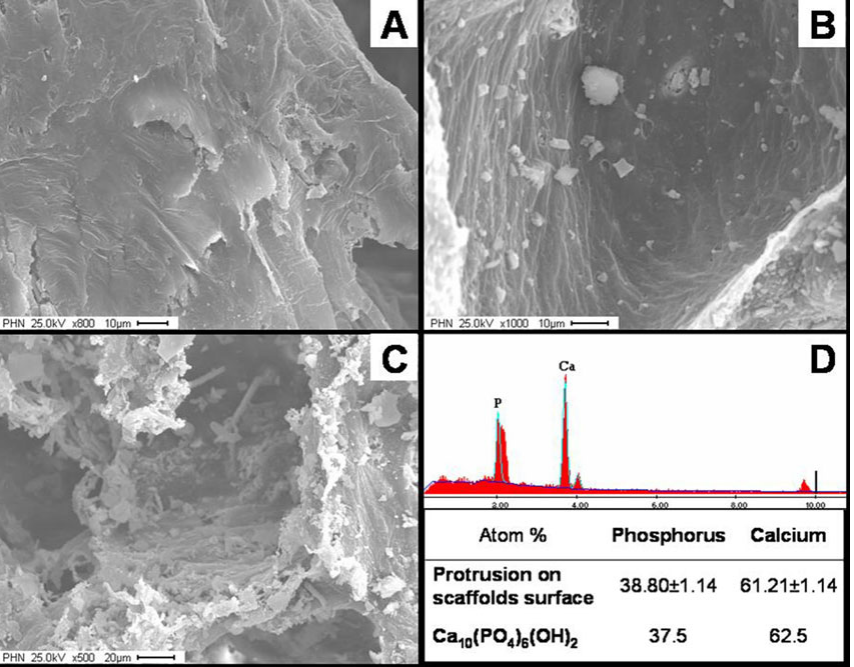
**Microstructure of scaffolds exhibited by SEM**. The HA were embedded in PCL, or exposed on the surface. Apparently the roughness of pore-wall surfaces increased with increasing the HA ratio. Panel (A) (800×) was an example of HA-free PCL scaffolds. Panel (B) (1000×) showed composite with low HA ratio, HA: PCL (*w/w*) = 1:4; while Panel (C) (500×) revealed a sample with high HA concentration, HA: PCL (*w/w*) = 1:1. The protruded components were confirmed as the HA by EDX (Panel D). The peaks of calcium and phosphorus were prominent and quantified the atomic ratio (Ca: P = 1.58).

PCL scaffolds incorporated with or without HA were fabricated with controlled porosity (70% ~ 75%) and pore sizes. Interconnected pore morphologies were present in all scaffolds, resulting in the high porosity of the scaffolds. Different microtopographies of scaffolds were revealed by scanning electron microscopy (SEM) (Figure 4). The roughness of pore wall appeared dependent on the ratio of HA to PCL. High HA concentration led to extensive protrusions of HA particles and rough surfaces (Figure 4C), while an almost smooth pore wall was achieved in the PCL scaffold without HA incorporation (Figure 4A). EDX analysis indicated atomic ratio of calcium to phosphorus (Ca: P = 1.58) on both low HA ratio (HA: PCL = 1:4) and high HA ratio (HA: PCL = 1:1) composite scaffolds, which is comparable to a natural hydroxyapatite (Figure 4D).

**Figure 5:**
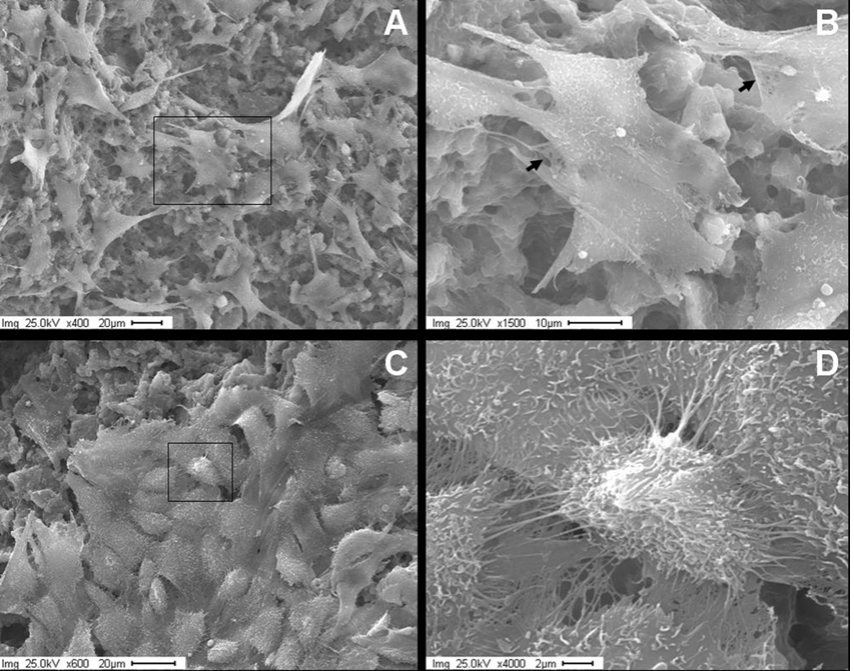
**Cells morphology on the composite scaffolds (HA: PCL = 1:1)**. Panel (A) showed osteoblasts proliferating on PCL-HA scaffolds (400×). Panel (B) was an enlargement of Panel (A) showing the details of the attached osteoblasts with meshwork of extracellular matrix (arrows), and cellular projection (1500×). Panels (C) and (D) revealed the growth of endothelial cells on a PCL-HA scaffold. The Panel (C) pictured ECs proliferating and forming a cell sheet (600×), and Panel (D) magnified attached ECs to detail their cobblestone shape and cellular extensions (4000×).

The specific cell morphology of either osteoblasts or endothelial cells displayed rarely difference response to various groups of PCL-HA scaffold. The continuous culture of osteoblasts on HA-containing scaffolds for 7 days revealed that cells retained their spindle morphology (Figure 5A) similar to the osteoblasts grown on tissue culture flasks. Extracellular matrix (collagen-like fibers) was clearly present at intercellular regions, where cellular projections were evident (Figure 5B). For the cultures of PCL-HA scaffolds with bone marrow-derived endothelial cells, the cells completely covered the surface of the scaffolds at 7 days (Figure 5C). The spreading and paving endothelial cells remained as typically cobblestone like shapes with high cell-to-cell contact (Figure 5D). Cellular extensions on the cells surface were also detected.

**Figure 6:**
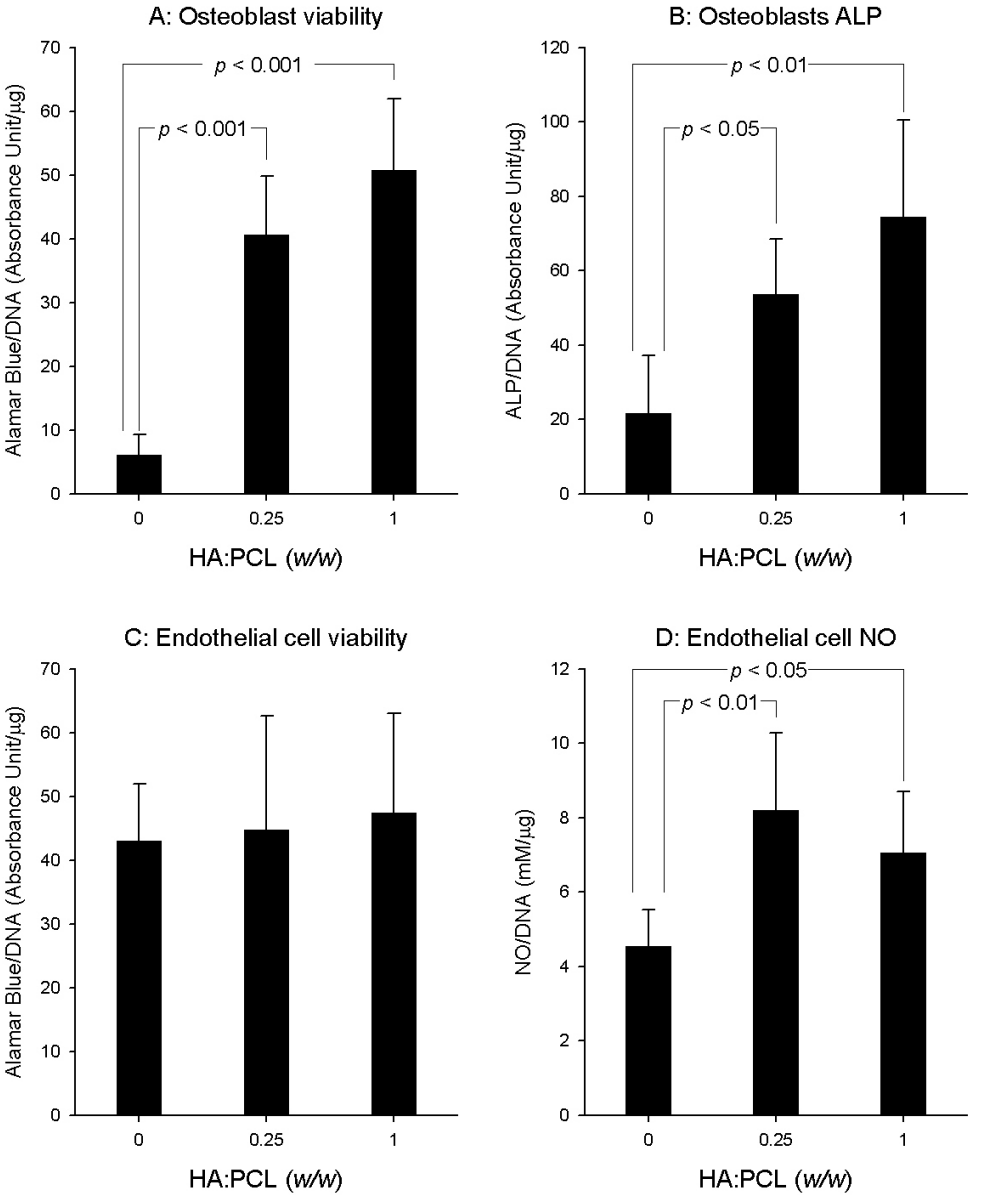
**Effects of composite scaffolds on the cells viability and function**. The induced osteoblasts and endothelial cells were separately cultured with the 3 groups of scaffold (30 scaffolds per group for each type of cell) for 7 days before testing. Panels (A) and (C) summarized the Alamar Blue assay for osteoblast and endothelial cell viabilities. Panel (B) plotted alkaline phosphatase activity of osteoblasts on various scaffolds. Panel (D) showed the NO production of endothelial cells on various scaffolds.

The biochemical tests would provide quantitive comparison on compatibility of biomaterials across various groups. Comparisons were made among cell-scaffold complexes that varied only in HA ratio to PCL. Addition of HA resulted in a positive stimulation of osteoblasts viability (Figure 6A). Compared to the HA-free PCL material, osteoblasts viability was increased by 740% (*p *< 0.001) in high HA ratio (HA: PCL = 1:1) group and by 570% (*p *< 0.001) in low HA ratio (HA: PCL = 1:4) group, revealed by the Alamar Blue assay. Similarly, as a marker of osteoblast differentiation, ALP activity was increased by 240% in the high HA ratio group (*p *< 0.01) and by 150% in low HA ratio group (*p *< 0.05), suggesting the promotion of osteoblasts function due to the osteoconductivity of HA (Figure 6B). There were no statistically significant difference between the two HA-containing groups in terms of osteoblasts viabilities (*p *= 0.083) and ALP activities (*p *= 0.119). Although the addition of HA into PCL did not show significant influence on endothelial cell viability (Figure 6C), the HA involvement into PCL did lead to 36% (at HA: PCL = 1:1) (*p *< 0.05) and 80% (at HA: PCL = 1:4) (*p *< 0.01) increase of nitric oxide production compared to the HA-free scaffolds (Figure 6D). There was not statistically significant difference on NO production between low and high HA ratio groups (*p *= 0.292, Figure 6D).

## Discussion

Bone tissues are essentially composite materials consisting of various components tissues with different structural arrangements and functions. Repairing bone defects using tissue engineered grafts involves the interplay of several variables, including scaffold materials with excellent biocompatibility, osteogenic cells capable of assembling the complicated and functional bone tissue, and an appropriate vascular bed to support the metabolic needs of new bone tissue. We have been developing a composite scaffold consisting of PCL and HA to enhance the biomechanical properties as the potential bone graft substitute [19]. Furthermore, following introducing MSCs-derived osteoblast (responsible for osteogenesis) and EPCs-derived endothelial cells (accountable for vascularization), we attempted in the current study to evaluate the biological impacts of various component ratios in PCL-HA scaffolds on the two types of cells.

Large numbers of MSCs-originated osteoblasts and EPCs-originated endothelial cells could be obtained in dish culture *in vitro*, making them possible to construct transplantable tissues scaffolds. It has been shown that *in vitro *stem/progenitor cells possess the capability of self-renewal and differentiation into organ-specific cell types [20]. Our results indicated that MSCs and EPCs could be harvested from mice bone marrow and differentiate into osteoblasts and endothelial cells respectively. Besides the expression of specific molecular markers like osteocalcin, VEGFR-2, and vWF, induced MSCs and EPCs functioned normally, as indicated by both fine crystals of ossification (comparable to natural bone mineral) and ALP activity in osteoblasts, and capillary-like formation by endothelial cells in Matrigel™. Due to the capacity of capillary formation, it was speculated that induced endothelial cells might participate or mediate the neovascularization in tissue engineered bone *in vivo*, which had been elucidated in a study [15]. It demonstrated that these differentiated bone marrow-derived endothelial cells and osteoblasts had the potential to create a tissue-engineered bone graft with microvascular network. Therefore, it is necessary to evaluated biological effects PCL-HA scaffolds on these cells seeded on it.

PCL and HA have been shown non-toxic and non-mutagenic biomaterials [11,21-24]. In particular, the biocompatibility of HA due to its similarities to natural bone mineral have led to HA widespread use in bone reconstructive surgery. Degradation and metabolism of PCL was completed *in vivo*, and ε-hydroxycaproic acid and water were the only metabolites [22,25]. Our study indicated that inclusion of HA into PCL significantly increased the mitochondrial activity and expression of ALP by osteoblasts in a dose-dependent manner, which is in agreement with previous studies [12,26]. The SEM images demonstrated that bone marrow-derived osteoblasts were spread on the scaffold surfaces with exposed HA particles and synthesized extracellular matrix. Nitric oxide (NO) is a biologically active molecule in the maintenance of vascular homeostasis and predominately produced by endothelial cells. We examined the levels of NO in cell media and in cell-scaffold composite to confirm and quantify the function of bone marrow-derived endothelial cells. Our data showed that the addition of HA elevated NO production in comparison with the HA-free PCL scaffolds, suggesting that HA particles promoted functions of the endothelial cells. Although Pezzatini [27] reported excellent biocompatibility of HA nanocrystals for endothelial cells, cell viability experiments of this study resulted no difference among endothelial cell groups. The different culture conditions and cell origins, disparate HA dimensions, and biomaterials architectures between our and Pezzatini's investigations may partially explain the diverse outcomes.

In this study, adding of HA into PCL led to heterogeneous surface properties. Variety of HA ratio to PCL generated distinct exposure of HA particle and diverse topography, which have been evidenced by SEM images. The exposed HA particles were more predominated on the high HA ratio scaffold than those on the lower ones, where the HA free scaffold just exhibited smoother surface on the pore walls. It appears that the rough surfaces due to HA embedding provided more anchorage for cell process and spreading, adhesion and orientation. Therefore, osteoblasts and endothelial cells were inclined to attach to the rougher surface and appeared better viability and strong cellular phenotypes, which supplement the literature reports that cell number and attachment force were increased on textured polymer substrates [26,28,29]. Furthermore HA is a well known sorbent for molecules. Involvement of HA particles on the PCL surface may change the surface charge, reduce the hydrophobicity of PCL and promote the adsorption of proteins and other molecules from surrounding environment [30,31]. In hard tissues, proteins such as osteopontin, bone sialoprotein, and osteocalcin were able to recognize HA through highly acidic domains, resulting in the attachment and distribution of osteogenic cells on the surface of protein-coated HA, and subsequently improving cell proliferation and differentiation, and promoting new bone formation [32-35]. Additionally the calcium ions released from the dissolution of HA were able to neutralize PCL acidic products, so the adverse response due to the PCL degradation could be overcame [14,36,37].

## Conclusion

In conclusion, our data indicated that supplement of HA into PCL provided a compatible environment for osteoblasts and endothelial cells to replicate and function. The HA surface exposure accounted for the positive cellular responses. Optimal component ratio in PCL-HA scaffold could be selected in term of bioactivities of osteoblasts and endothelial cells. These outcomes would contribute to the construction of vascularized engineered bone in vitro and implantation *in vivo*.

## Competing interests

The authors declare that they have no competing interests.

## Authors' contributions

HY, PHW and SYY contributed to the design of the study and the writing of the manuscript. HY carried out the cell culture, scaffold preparation, biochemistry assessment, acquisition, and analysis of data. SYY participated in the image and statistical analysis. All authors read and approved the final manuscript.
